# Rapid inactivation of SARS-CoV-2 with deep-UV LED irradiation

**DOI:** 10.1080/22221751.2020.1796529

**Published:** 2020-07-31

**Authors:** Hiroko Inagaki, Akatsuki Saito, Hironobu Sugiyama, Tamaki Okabayashi, Shouichi Fujimoto

**Affiliations:** a M&N Collaboration Research Laboratory, Department of Medical Environment Innovation, Faculty of Medicine, University of Miyazaki, Miyazaki, Japan; b Department of Veterinary Science, Faculty of Agriculture, University of Miyazaki, Miyazaki, Japan; c Center for Animal Disease Control, University of Miyazaki, Miyazaki, Japan; d Nikkiso Co., LTD, Tokyo, Japan; e Department of Hemovascular Medicine and Artificial Organs, Faculty of Medicine, University of Miyazaki, Miyazaki, Japan

**Keywords:** COVID-19, deep-UV LED, inactivation, SARS-CoV-2, fomite infection, contact transmission, antiviral efficacy

## Abstract

The spread of novel coronavirus disease 2019 (COVID-19) infections worldwide has raised concerns about the prevention and control of SARS-CoV-2. Devices that rapidly inactivate viruses can reduce the chance of infection through aerosols and contact transmission. This *in vitro* study demonstrated that irradiation with a deep ultraviolet light-emitting diode (DUV-LED) of 280 ± 5 nm wavelength rapidly inactivates SARS-CoV-2 obtained from a COVID-19 patient. Development of devices equipped with DUV-LED is expected to prevent virus invasion through the air and after touching contaminated objects.

## Letter

The novel coronavirus SARS-CoV-2 pandemic has spread worldwide and placed countries in emerging, rapidly transforming situations. More than 5.3 million cases of COVID-19 and 342,000 deaths had been reported to WHO by 25 May 2020 [1]. Infectious virus is detected in specimens from the respiratory tract, nasopharyngeal sites, and feces in COVID-19 patients [2]. Recently, infectious SARS-CoV-2 was isolated from the urine of a COVID-19 patient [3]. SARS-CoV-2 is detectable in aerosols for up to 3 h, up to 4 h on copper, up to 24 h on cardboard and up to 2–3 days on plastic and stainless steel [4]. In nasal mucus and sputum, this virus is undetectable after 48 h, but it’s RNA remained detectable for 7 days [5]. To prevent exposure to contaminated material (fomite infection) which appears to be an important transmission route after droplet infection, hand hygiene with alcohol is key and efficient, but additional measures in preventing the spread of SARS-CoV-2 infection may be needed [6,7].

A deep ultraviolet light-emitting diode (DUV-LED) instrument generating around 250–300 nm wavelength has been reported to effectively inactivate microorganisms, including bacteria, viruses and fungi [8–11], but effects on SARS-CoV-2 have not been reported. We evaluated the antiviral efficacy of irradiation by DUV-LED, generating a narrow-range wavelength (280 ± 5 nm, mean ± SD) (Nikkiso Co., Tokyo, Japan), which we tested against SARS-CoV-2. This wavelength was selected due to higher output (radiation) power and longer lifetime in the stage of LED development considering practicality.

A strain of SARS-CoV-2 isolated from a patient who developed COVID-19 in the cruise ship *Diamond Princess* in Japan in February 2020 [12] was obtained from the Kanagawa Prefectural Institute of Public Health (SARS-CoV-2/Hu/DP/Kng/19-027, LC528233). The virus was propagated in Vero cells cultured in minimum essential medium (MEM) containing 2% fetal bovine serum (FBS). At 48 h after infection, virus stocks were collected by centrifuging the culture supernatants of infected Vero cells at 3000 rpm for 10 min. Clarified supernatants were kept at −80°C until use. Aliquots of stock virus were diluted with phosphate-buffered saline and adjusted to 2.0 × 10^4^ plaque-forming units (PFU)/ml. For the evaluation of DUV-LED inactivation, aliquots of virus stock (150 μl) were placed in the centre of a 60-mm Petri dish and irradiated with 3.75 mW/cm^2^ at work distance 20 mm for a range of times (n = 3 each time for 1, 10, 20, 30, or 60 s, and each dose corresponding to 3.75, 37.5, 75, 112.5 or 225 mJ/cm^2^, respectively). After irradiation with DUV-LED, approximately 120 μl of each virus stock was collected with a 200 μl tip. Virus solutions were serially diluted in 10-fold steps using serum free MEM in a 1.5 ml tube, then inoculated onto Vero monolayers in a 12-well plate. After adsorption of virus for 2 h, cells were overlaid with MEM containing 1% carboxymethyl cellulose and 2% FBS (final concentration). Cells were incubated for 72 h in a CO_2_ incubator, then cytopathic effects were observed under a microscope. An unirradiated virus suspension was used as a negative control. To calculate PFU, cells were fixed with 10% formalin for 30 min, followed by staining with 0.1% methylene blue solution. The antiviral effects of DUV-LED irradiations were assessed using the logPFU ratio, calculated as logPFU ratio = log_10_ (Nt/N0), where Nt is the PFU count of the UV-irradiated sample, and N0 is the PFU count of the sample without UV irradiation. In addition, the infectious titer reduction rate was calculated as (1–1/10^log PFU ratio^) × 100 (%). All experiments were performed in a BSL-3 laboratory.

**Figure 1.**
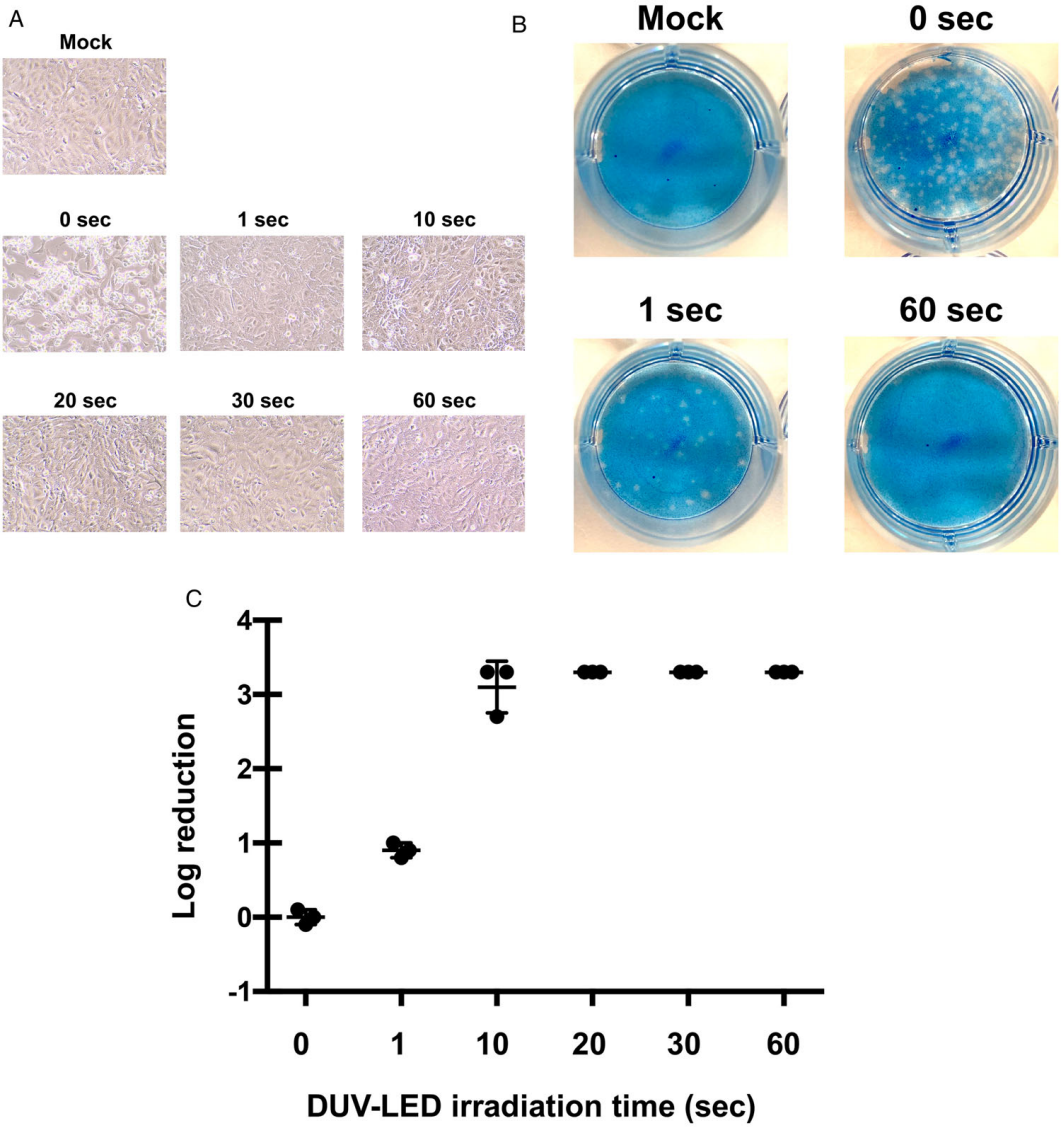
Inhibitory effects of DUV-irradiation on SARS-CoV-2. (A) Cytopathic changes in virus-infected Vero cells without DUV-LED irradiation (0 s), or with DUV-LED irradiation for 1, 10, 20, 30 or 60 s, and each dose corresponding to 3.75, 37.5, 75, 112.5 or 225 mJ/cm^2^, respectively. (B) Plaque formation in Vero cells. Virus solutions irradiated with DUV-LED for several durations were diluted (100-fold) and inoculated to Vero cells. A representative result is shown. (C) Time-dependent inactivation of SARS-CoV-2 by DUV-LED irradiation. The results shown are the mean and standard deviation (SD) of triplicate measurements.

We observed a marked cytopathic effect in virus-infected cells without DUV-LED irradiation (Figure 1A, see “0 s”). In contrast, virus-infected cells irradiated for 60 s showed largely comparable morphology to mock cells (Figure 1A, see “60 s”). To our surprise, the cells inoculated with virus irradiated for 1 s looked similar to mock cells with minimal cytopathic effects (Figure 1A, see “1 s”). The plaque assay (Figure 1B) revealed that short time DUV-LED irradiation rapidly inactivated SARS-CoV-2 (Figure 1C and Table 1). Of note, the infectious titer reduction rate of 87.4% was already recognized with irradiation of virus stock for 1 s, and the rate was 99.9% with irradiation for 10 s. These results suggest that DUV-LED drastically inactivated SARS-CoV-2 with irradiation for even a very short time.

**Table 1.** Differences in infectious titer with different DUV-LED irradiation times.

Irradiation time	Control (no irradiation)	DUV-LED irradiation time				
		1 s	10 s	20 s	30 s	60 s
PFU (PFU/mL)	3.7 × 10^4^	4.7 × 10^3^	2.7 × 10^1^	6.7 × 10^0^	<20	<20
Log PFU ratio^a^	–	0.9	3.1	>3.3	>3.3	>3.3
Infection titer reduction ratio^b^ (%)	–	87.4	99.9	>99.9	>99.9	>99.9

^a^log_10_ (Nt/N0) where Nt is the PFU count of the UV-irradiated sample and N0 is the PFU count of the sample without UV irradiation.

^b^(1–1/10^log PFU ratio^) × 100 (%).

UV-LEDs providing irradiation at various peak emission wavelengths, such as UV-A (320–400 nm), UV-B (280–320 nm), and UV-C (100–280 nm), have been adopted to inactivate various pathogenic species, including bacteria, viruses and fungi. Devices equipped with UV-LEDs are now beginning to be introduced into medical fields. UV-C is considered to be the most effective germicidal region of the UV spectrum, acting through the formation of photoproducts in DNA and RNA [13]. These pyrimidine dimers interrupt transcription, translation and replication of DNA and RNA, eventually leading to inactivation of microorganisms [14]. The efficacy of this inactivation may depend not only on the wavelength, but also on factors such as the target (e.g. bacterial species), light output and environmental conditions. The DUV-LED we used has the characteristics that can irradiate a narrow-range wavelength (280 ± 5 nm) compared with ordinary lamp, and achieve both long lifetime and high output power in time. This study demonstrated for the first time the rapid inactivation of SARS-CoV-2 under DUV-LED irradiation. As shown in Figure 1A, cytopathic effects were observed in control Vero cells infected with SARS-CoV-2, but not in the cells with DUV-LED irradiation for only 10 s. Whether UV-LED can similarly inactivate SARS-CoV-2 viruses existing in bodily fluid such as saliva needs to be investigated in the future. As well as in community settings, healthcare settings are also vulnerable to the invasion and spread of SARS-CoV-2, and the stability of SARS-CoV-2 in aerosols and on surfaces [4] likely contributes to virus transmission in medical environments. Although various monoclonal antibodies and clinical trials showing some results with first drugs (remdesivir) have been published [15], no vaccines are currently available for prevention and treatment of SARS-CoV-2. By revealing that SARS-CoV-2 inactivation can be achieved with very short-term DUV-LED irradiation, this study provides useful baseline data toward securing a safer medical environment. In the future, it will be necessary to validate the relationship between the radiation distance by DUV-LED and the inactivation of target virus. Development of devices equipped with DUV-LED is expected to prevent the virus invasion through the air and after touching contaminated objects.
